# Reproducible spectral CT thermometry with liver-mimicking phantoms for image-guided thermal ablation

**DOI:** 10.1088/1361-6560/ad2124

**Published:** 2024-02-05

**Authors:** Leening P Liu, Rizza Pua, Derick N Rosario-Berrios, Olivia F Sandvold, Amy E Perkins, David P Cormode, Nadav Shapira, Michael C Soulen, Peter B Noël

**Affiliations:** 1 Department of Radiology, University of Pennsylvania, Philadelphia, PA, United States of America; 2 Department of Bioengineering, University of Pennsylvania, Philadelphia, PA, United States of America; 3 Department of Biochemistry and Biophysics, University of Pennsylvania, Philadelphia, PA, United States of America; 4 Philips Healthcare, Orange Village, OH, United States of America

**Keywords:** spectral computed tomography, computed tomography, thermometry, CT thermometry, thermal ablation, interventional radiology, microwave ablation

## Abstract

*Objectives*. Evaluate the reproducibility, temperature tolerance, and radiation dose requirements of spectral CT thermometry in tissue-mimicking phantoms to establish its utility for non-invasive temperature monitoring of thermal ablations. *Methods*. Three liver mimicking phantoms embedded with temperature sensors were individually scanned with a dual-layer spectral CT at different radiation dose levels during heating (35 °C–80 °C). Physical density maps were reconstructed from spectral results using varying reconstruction parameters. Thermal volumetric expansion was then measured at each temperature sensor every 5 °C in order to establish a correlation between physical density and temperature. Linear regressions were applied based on thermal volumetric expansion for each phantom, and coefficient of variation for fit parameters was calculated to characterize reproducibility of spectral CT thermometry. Additionally, temperature tolerance was determined to evaluate effects of acquisition and reconstruction parameters. The resulting minimum radiation dose to meet the clinical temperature accuracy requirement was determined for each slice thickness with and without additional denoising. *Results*. Thermal volumetric expansion was robustly replicated in all three phantoms, with a correlation coefficient variation of only 0.43%. Similarly, the coefficient of variation for the slope and intercept were 9.6% and 0.08%, respectively, indicating reproducibility of the spectral CT thermometry. Temperature tolerance ranged from 2 °C to 23 °C, decreasing with increased radiation dose, slice thickness, and iterative reconstruction level. To meet the clinical requirement for temperature tolerance, the minimum required radiation dose ranged from 20, 30, and 57 mGy for slice thickness of 2, 3, and 5 mm, respectively, but was reduced to 2 mGy with additional denoising. *Conclusions*. Spectral CT thermometry demonstrated reproducibility across three liver-mimicking phantoms and illustrated the clinical requirement for temperature tolerance can be met for different slice thicknesses. The reproducibility and temperature accuracy of spectral CT thermometry enable its clinical application for non-invasive temperature monitoring of thermal ablation.

**Abbreviations**
CT: computed tomography
HU: Hounsfield Units
VMI: virtual monoenergetic images
ROI: regions of interest
FBG: fiber Bragg grating
CTDI_vol_
: volumetric CT dose index
IR: iterative reconstruction
R: Pearson’s correlation coefficient
CV: coefficient of variation

## Introduction

Thermal ablation has become increasingly utilized for minimally invasive treatment of tumors in patients with low disease burdens as an alternative to surgical resection (Petrowsky and Busuttil 2008, Uhlig *et al* 2018). In each of the different methods of thermal ablation (radiofrequency, microwave, laser, high intensity focused ultrasound), an ablation applicator focally heats tumor tissue and a surrounding safety margin to a lethal threshold of 60 °C that induces cell death within seconds (Wright *et al* 1998, Goldberg *et al* 2000). Even with improvements in ablation technology, local tumor recurrence rates remain undesirably high, ranging from 2% to 17% for microwave ablation (Yin *et al* 2009, Zhou *et al* 2009, Bhardwaj *et al* 2010, Groeschl *et al* 2013, Liu *et al* 2013, Vietti Violi *et al* 2018) compared to 9% to 37% for radiofrequency ablation, an older technology (Aloia *et al* 2006, Abitabile *et al* 2007, Veltri *et al* 2008, Liu *et al* 2013, Abdelaziz *et al* 2014, Vietti Violi *et al* 2018). These local recurrences are strongly associated with an insufficient coverage of the tumor and the ablative safety margin around the tumor (Wang *et al* 2013, Teng *et al* 2015, Sotirchos *et al* 2016). Both can be assessed with visual/cognitive registration of the tumor on pre-ablation unenhanced CT and the estimated ablation zone on contrast-enhanced CT acquired immediately post-ablation (Wood *et al* 2007, Park *et al* 2008). With the addition of ablation confirmation software, automated registration has helped to identify tumors with insufficient margins, thus spurring additional treatment and increasing the percent of treated tumors with a sufficient margin from 61% to 77% (Joo *et al* 2022). Despite this increased effectiveness, necrotic tissue on contrast-enhanced CT immediately post-ablation is not only hard to distinguish from tumor tissue (Bressem *et al* 2019) but continues to expand at least 24 hours after the procedure, thus is not predictive of total cell death (Vogel *et al* 2019). Comparatively, using intra-tissue temperature as a predictor offers strong guidance for delineating the ablation zone. Furthermore, it can be monitored in real-time during the procedure to provide feedback to the interventional radiologist, potentially increasing the success of the ablation, reducing the risk of local tumor recurrence, and decreasing the risk of non-target injury to adjacent vulnerable structures, such as the pancreas and bowel.

Intraprocedural monitoring of temperature requires the generation of differential temperature maps to determine whether tissue temperature reaches and surpasses the lethal threshold. These temperature maps can be generated from conventional CT images through CT thermometry. CT thermometry simplifies thermal volumetric expansion to establish a linear, quadratic, or cubic relationship between changes in temperature and Hounsfield Units (HU) (Fallone *et al* 1982, Schena *et al* 2013). In the past few decades, CT thermometry has been evaluated in *ex vivo* tissues with different heating methods and CT scanners with a range of temperature sensitivities from −2.0 to −0.23 HU/°C (Fallone *et al* 1982, Pandeya *et al* 2011, Bruners *et al* 2012, Schena *et al* 2013, Pohlan *et al* 2020). The variability in tissue characteristics and scanner performance, which leads to a wide range in temperature sensitivity, coupled with the necessity for high radiation doses, has hindered the clinical implementation of this method. Advancements of CT, specifically spectral CT, have enabled novel and improved methodologies that utilize spectral results, which are less dependent on scanner technology and patient habitus (Hua *et al* 2018, Sauter *et al* 2018, Hokamp *et al* 2020). With dual-energy CT, the first realization of spectral CT, we previously developed and validated spectral CT thermometry (Liu *et al* 2023a). It uses spectral results to calculate physical density maps that relate to temperature through thermal volumetric expansion (Liu *et al* 2023a). Compared to traditional methods that rely on HU, which can reflect both changes in temperature and changes in tissue composition, the use of physical density maps isolates changes in temperature, ensuring more accurate temperature mapping (Liu *et al* 2023a). The resulting strong correlation to thermal volumetric expansion demonstrated the feasibility of spectral CT thermometry and highlighted its potential utility for non-invasive temperature monitoring.

In order to progress towards *in vivo* evaluation and clinical translation, open questions about reproducibility and clinical requirements of spectral CT thermometry must be addressed. With the large range in model parameters in previously published studies of CT thermometry, reproducibility of the model with the same tissue/object is especially important to establish confidence in the method (Pohlan *et al* 2020). Similarly, temperature accuracy of <3 °C and an in-plane spatial resolution of <2 mm are required to ensure that detected temperatures at a specific voxel correspond to true tissue temperatures, and consequently ablation success (Frich 2006). In particular, the temperature accuracy requirement is uniquely intertwined with radiation dose because of noise. Noise in physical density maps decreases with radiation dose, thus improving temperature tolerance crucial for temperature map accuracy. Specifically, tissue heterogeneity in our previous study masked physical density noise (Liu *et al* 2023a), suggesting the need for reproducible and homogeneous phantoms for evaluation of this tradeoff as well as reproducibility of the model.

This study aimed to assess the reproducibility of spectral CT thermometry with liver-mimicking gel phantoms. We also examined the effect of radiation dose and reconstruction parameters on temperature tolerance, both at baseline and with additional denoising, and determined the corresponding radiation dose required to achieve acceptable temperature tolerance. Our results demonstrated strong reproducibility and sufficiently low temperature tolerance and radiation doses, supporting the clinical translation of spectral CT thermometry for non-invasive temperature monitoring during thermal ablations.

## Materials and methods

### Phantom synthesis and characterization

To evaluate reproducibility of spectral CT thermometry and its temperature tolerance for liver ablation via thermal volumetric expansion, a homogeneous tissue-mimicking phantom as described in Negussie *et al* was modified to mimic the attenuation of liver tissue (Negussie *et al* 2016). It consists of a homogenous gel with a diameter of 8 cm and a height of 10.5 cm and replicated the attenuation and thermal properties of liver. Briefly, 1 g of ammonium persulfate (Sigma Aldrich, St. Louis, Missouri) was dissolved in 2 ml of deionized water and then vortexed and sonicated until homogeneous. Separately, 290 ml of degassed and deionized water and 200 ml of 40% acrylamide/bisacrylamide (Sigma Aldrich, St. Louis, Missouri) were mixed by magnetic stirring. The ammonium persulfate solution and 1 ml of N,N,N’,N’-tetramethylethylenediamine (Sigma Aldrich, St. Louis, Missouri) were then added in quick succession to start and catalyze polymerization, respectively. The solution was then placed in a tightly sealed plastic container and placed at 4 °C for an hour. The phantom was then moved to room temperature to continue to solidify until use.

While mass density, tissue conductivity, and thermal diffusivity of the original phantom were similar to that of human soft tissues, the attenuation profile did not match liver as described by ICRU 46 Report (ICRU 1992). As a result, varying amounts of calcium chloride dihydrate (Sigma Aldrich, St. Louis, Missouri) were added until the profile was adequately equivalent to the expected attenuation of liver tissue between 40 and 200 keV (± 5 HU). Each modified phantom was evaluated by scanning the phantom on a dual-layer spectral CT (IQon Spectral CT, Philips Healthcare, Eindhoven, Netherlands) at a tube voltage of 120 kVp. Full scanning and reconstruction parameters are in table 1. Virtual monoenergetic images (VMI) were then collected every 10 keV between 40 and 200 keV, and regions of interest (ROI) were placed in the center of the phantom to measure attenuation. The resulting spectral curve was compared to the corresponding spectral curve for human liver from ICRU 46 (ICRU 1992). Ultimately, to match the attenuation curve, 5.8 g of calcium chloride dihydrate (Sigma Aldrich, St. Louis, Missouri) was dissolved in 10 ml of deionized water and added to the acrylamide and deionized water solution prior to polymerization. Additionally, tissue conductivity, volumetric heat capacity, and thermal diffusivity were measured with a thermal analyzer (Tempos Thermal Properties Analyzer, Meter Environment, Pullman, Washington). After confirmation of these phantom properties, the phantom was deemed liver-mimicking and sufficiently accurate for spectral CT thermometry of liver.

**Table 1.** Acquisition and reconstruction parameters.

	Phantom composition	Thermometry
Tube voltage [kVp]	120	
Exposure time [s]	0.75	
CTDIvol [mGy]	20	2, 5, 10, 20, 30, 56.8
Collimation width [mm]	64 × 0.625	
Slice thickness [mm]	2	2, 3, 5
Convolution kernel	B	A, B
iDose level	0	0, 2, 4, 6
Field of view [mm]	350	
Matrix size	512 × 512	
Pixel spacing [mm]	0.68	

For thermometry experiments, a phantom was prepared two to three days prior to each of the three repetitions. Before phantom synthesis, a 3D printed guide was glued to the lid of the plastic container to help guide two temperature probes for ground truth measurements. A fiber Bragg grating (FBG) temperature sensor (FiSpec FBG X100, FiSens GmbH, Braunschweig, Germany) was mounted 2 cm from the center and contains four sensors along its length. The four sensors are 1 mm in length and spaced 1 cm from each other along the fiber. An optical fiber sensor (Fiber Optic Temperature Sensor, Omega Engineering, Norwalk, Connecticut) was also placed 3 cm from the center and only measures temperature at the tip of the fiber. In total, temperature was measured at five different locations. Both fibers were inserted into the phantom immediately after polymerization was initialized to ensure that they were aligned and embedded.

### Image acquisition

**Figure 1.**
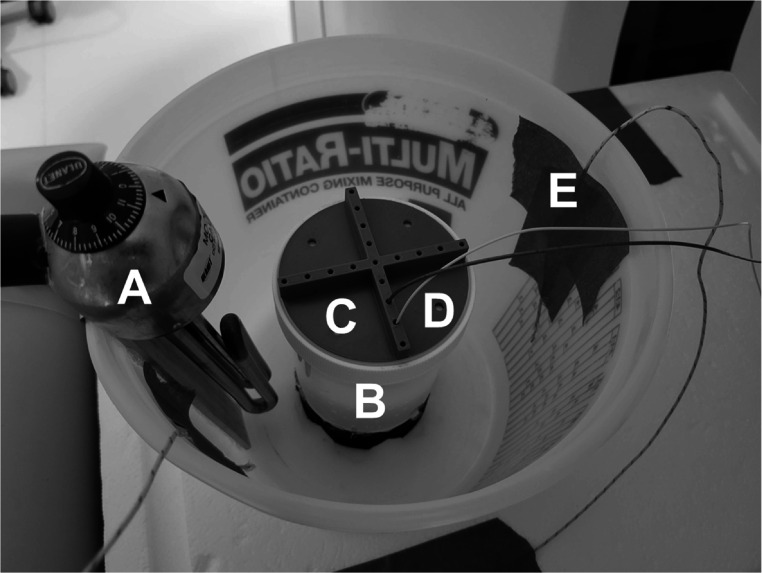
**Experimental setup for reproducibility of spectral CT thermometry**. Hot water and an immersion heater (A) were utilized to heat a liver-mimicking phantom (B). A 3D printed guide (C) helped accurately place thermometers (D), a FBG grating fiber and optical fiber thermometer, to measure internal phantom temperatures. Additional thermocouples (E) were placed in the water to monitor temperatures.

**Figure 2.**
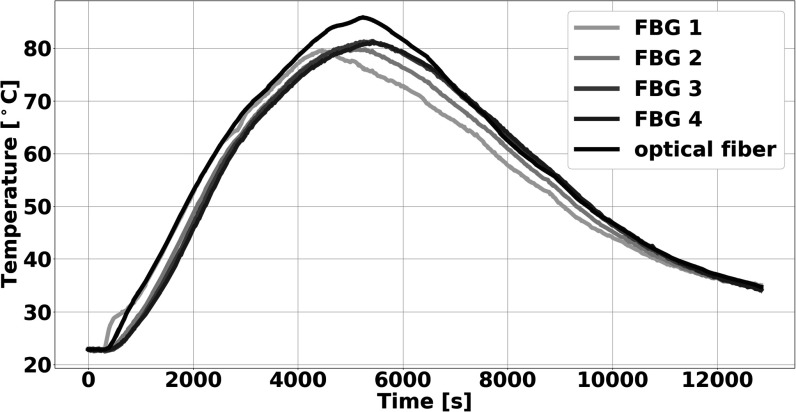
**Measured internal phantom temperature during heating and cooling of a single phantom**. Rise in temperatures occurred with heating from both hot water and an immersion heater, while cooling resulted from addition of ice into the water bath.

We assessed the reproducibility and temperature tolerance of spectral CT thermometry using three phantoms subjected to a range of temperatures in a water bath. Each liver-mimicking phantom was placed in a plastic bucket with an immersion heater (Heet-O-Matic Immersion Heater, Ulanet, Bristol, Connecticut) for continuously heating the water and two thermocouples (Type K Thermocouple, Pico Technology, St. Neots, United Kingdom) for monitoring the temperature of the water next to and across from the heater (figure 1). Boiled water was carefully poured into the bucket to completely immerse the phantom. After water temperatures cooled to 60 °C, the immersion heater was turned on to further heat the water until the phantom reached approximately 80 °C. Water temperatures rose on average 3.5 °C per minute. Then, ice was added to the water approximately every 20 min until the phantom cooled down to 35 °C (figure 2). During heating and cooling phases, the phantom was scanned with dual-layer spectral CT at a tube voltage of 120 kVp with axial scans and 4 cm collimation. Scans were performed approximately every 30 s, altering between different radiation doses at volumetric CT dose index (CTDI_vol_) of 2, 5, 10, 20, 30, and 56.8 mGy. Other acquisition parameters can be found in table 1. The process of heating, cooling, and scanning was repeated for each of the three separate phantoms to evaluate reproducibility.

**Figure 3.**
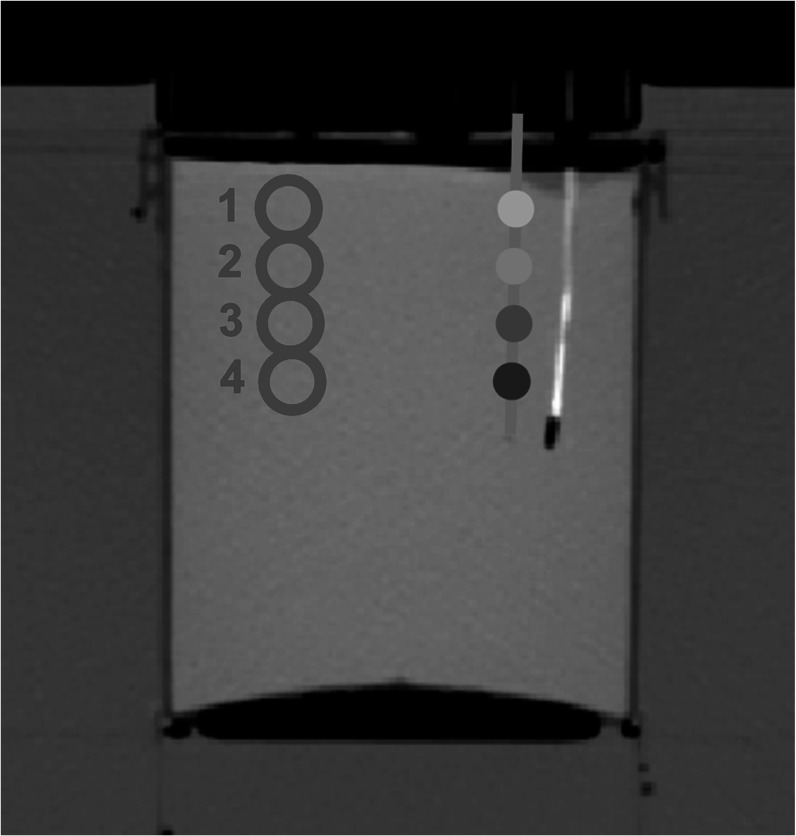
**Corresponding physical density measurement for each FBG along the fiber on axial images**. The high attenuating fiber corresponds to the optical fiber with a single sensor at its end while the blue line represents the FBG fiber with its sensors along its length (dots). ROIs (red circles) were placed symmetrically from each temperature sensor to avoid including thermometers in ROIs.

Axial physical density maps were utilized to relate physical density and temperature through thermal volumetric expansion. First, spectral results, specifically VMI at 70 keV and effective atomic number maps, were generated for acquisitions between approximately 35 °C and 80 °C at an interval of 5 °C for each radiation dose. Reconstructions varied in slice thickness (2, 3, 5 mm), iterative reconstruction (IR) level (iDose (Wright *et al* 1998) level 0, 2, 4, 6), and reconstruction kernel (A, B) to investigate the effect of parameters on temperature tolerance. These slice thicknesses, particularly 2 and 3 mm, are utilized clinically in our institution. Using VMI 70 keV and effective atomic number maps, physical density maps were calculated by applying a previously developed model (Liu *et al* 2023a). Then, in order to maximally decrease noise and thus decrease the minimum required dose for clinical translation, additional denoising with non-local means was also applied to spectral results prior to generating physical density maps by using the scikit-image Python package. Non-local means denoising uses a pixel-weighted average based on the similarity to the region (Buades *et al* 2005). From the reconstructed spectral results, physical density maps were generated by using a previously developed spectral physical density model. On these physical density maps, the tip of the fiber optic temperature sensor, the tip of the FBG fiber, and the angle of the FBG fiber were manually determined. With this information, locations of the five temperature sensors were calculated. ROIs were then placed opposite of the temperature sensors and equidistant to the center axis of the phantom to avoid the attenuation of the fibers (figure 3). Placement of these ROIs were possible because of the homogenous nature of the phantom and the left-right symmetry of the heating of the phantom. Mean and standard deviation of physical density at each temperature sensor were then recorded at each corresponding temperature and reconstruction parameter combination. Additional denoising and all analysis was performed with Python.

### Spectral CT reproducibility

For each of the three phantoms, thermal volumetric expansion was utilized to relate physical density to change in temperature:

\begin{eqnarray*}\rho \left(T\right)=\frac{{\rho }_{0}\left({T}_{0}\right)}{1+\alpha {\mathrm{\Delta }}T},\end{eqnarray*}

where $\rho $ is physical density at temperature $T,$ ${\rho }_{0}$ is the initial physical density at the initial temperature ${T}_{0},$ Δ$T$ is the difference in temperature, and $\alpha $ is the thermal volumetric expansion constant. Specifically, a linear regression was applied to the relationship between the inverse of the physical density and change in temperature for an IR level 0, reconstruction filter A, and slice thickness of 3 mm.

\begin{eqnarray*}\frac{{\rho }_{0}\left({T}_{0}\right)}{\rho \left(T\right)}=a+b{\mathrm{\Delta }}T.\end{eqnarray*}

The initial physical density and temperature were calculated as the mean physical density and temperature across radiation dose at approximately 35 °C. Corresponding slope, intercept, and Pearson’s correlation coefficient (R) were recorded for each temperature sensor and repetition. Mean and standard deviation of the model parameters were calculated. Variation of fit parameters was characterized by the resulting coefficient of variation (CV), or standard deviation divided by the mean. Additionally, scatter plots were used to illustrate the recapitulation of thermal volumetric expansion for each of the three repetitions.

### Temperature tolerance

Temperature tolerance, defined as the maximum error between computed and measured temperature, was determined for each combination of radiation doses and reconstruction parameters at the lowest and highest temperatures. By applying thermal volumetric expansion (equation (1)), temperature tolerance was calculated through error propagation:

\begin{eqnarray*}\delta T\equiv \frac{Si{g}_{err}}{slope}=\frac{{\frac{\delta \rho }{{\rho }^{2}}}_{\mathrm{1,2}}}{\frac{\unicode{x02206}\left(\frac{1}{\rho }\right)}{\unicode{x02206}T}}=\frac{\frac{1}{2}\left[\frac{\delta \rho \left({T}_{1}\right)}{{\rho \left({T}_{1}\right)}^{2}}+\frac{\delta \rho \left({T}_{2}\right)}{{\rho \left({T}_{2}\right)}^{2}}\right]}{\frac{\frac{1}{\rho \left({T}_{2}\right)}-\frac{1}{\rho \left({T}_{1}\right)}}{{T}_{2}-{T}_{1}}},\end{eqnarray*}

where ${si}{g}_{{err}}$ is the propagated error of the model, ${slope}$ is the slope of the thermometry model, and $\delta \rho $ is noise (standard deviation in an ROI) in physical density maps. As a result, temperature tolerance is greatly dependent on noise, which can be modulated with acquisition parameters, reconstruction parameters, and denoising algorithms. To first establish baseline temperature tolerance without external denoising, temperature tolerance was calculated for different acquisition and reconstruction parameters. These values were then represented as a heatmap, where each subsection corresponded to a radiation dose and slice thickness combination. Also, for each slice thickness, the minimum radiation dose required to achieve clinically relevant temperature tolerances was recorded. The same calculations were performed to illustrate the radiation dose reduction with additional post-processing (Liu *et al* 2023b).

## Results

### Phantom characterization

**Figure 4.**
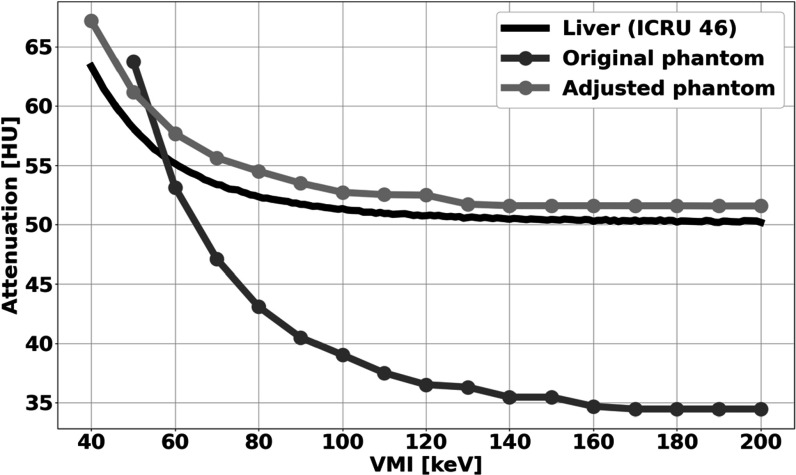
**Attenuation of liver-mimicking phantom across VMI energies**. Attenuation of the adjusted phantom better matched the attenuation of liver as described by ICRU 46 with a maximum difference of 4 HU.

The phantom was adequately modified to match the attenuation profile of human liver (figure 4). While the original phantom resulted in errors as great as 15 HU for different VMIs from 40 to 200 keV, the modified liver-mimicking phantom exhibited differences in attenuation to human liver tissue ranging of only up to 4 HU. Additionally, the thermal conductivity, thermal diffusivity, and volumetric heat capacity were 0.5209 W K^−1^ m^−1^, 0.18 mm^2 ^s^–1^, and 2.902 J m^−3^ K^−1^, respectively. These thermal properties corresponded to the thermal properties of human liver (Lopresto *et al* 2019).

### Spectral CT reproducibility

**Figure 5.**
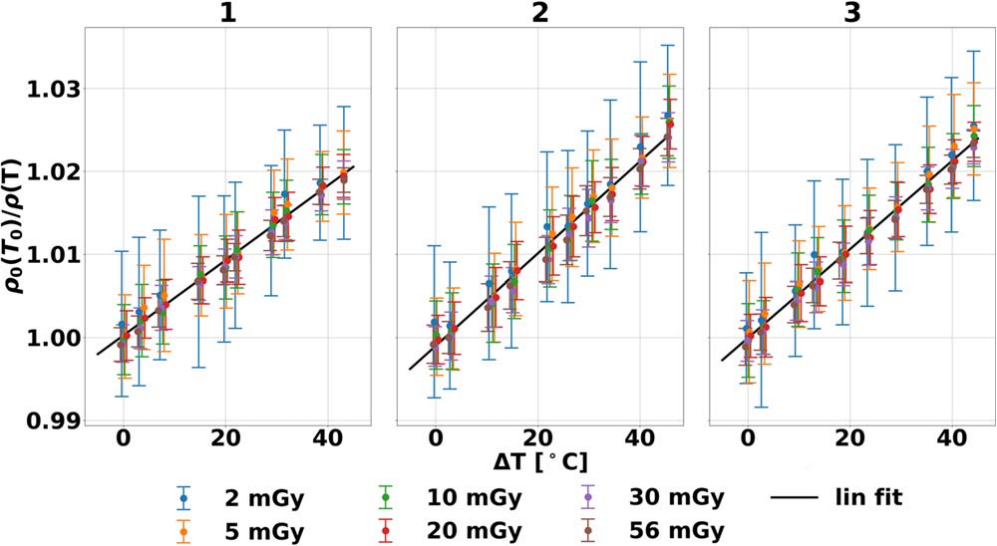
**Reproducibility of spectral CT thermometry across three phantoms between 35 and 80 °C**. The relationship between physical density and temperatures in three experiments exhibited linearity that corresponds to thermal volumetric expansion that is shown to be independent of dose. Additionally, similar behavior between the phantoms (numbered) highlighted the reproducibility of spectral CT thermometry.

Liver mimicking phantoms reflected a strong relationship between the inverse of the physical density and temperature that recapitulated thermal volumetric expansion for each repetition (figure 5). Slope and intercept measured 5.3 ± 0.5 × 10^−4^ °C^−1^ and 0.9995 ± 0.0008, respectively, across different repetitions, temperature sensors, and radiation doses without additional denoising. These values corresponded to a CV of 9.6% and 0.08% for slope and intercept, respectively, highlighting the reproducibility of spectral CT thermometry. The strong relationship was particularly demonstrated with an R of 0.989 ± 0.005 and a CV of 0.43%.

### Temperature tolerance

**Figure 6.**
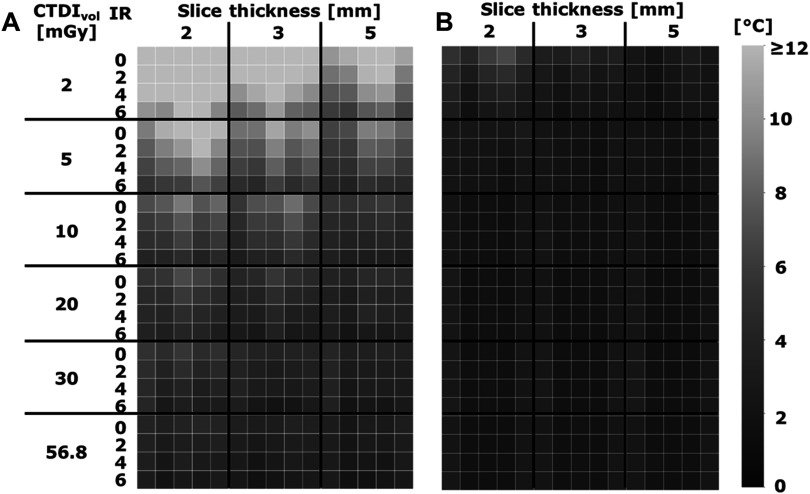
**Temperature tolerance of spectral CT thermometry**. In each subsection, the columns from left to right correspond to the different temperature sensors of the FBG and optical fiber. Temperature tolerance demonstrated a strong relationship with noise, improving with radiation dose, slice thickness, and IR level (A). Additional denoising further reduced temperature tolerance and reduced the effect of radiation dose and reconstruction parameters (B).

Temperature tolerance decreased with increased slice thickness, iterative reconstruction level, and radiation dose for each temperature sensor (figure 6(A)). Additionally, temperature tolerance was greater with reconstruction filter B than A. With reconstruction filter A, the smoothest filter, they ranged from 23 °C to 2.2 °C, 18 °C to 1.9 °C, and 14 °C to 1.8 °C for a slice thickness of 2, 3, and 5 mm, respectively. Specifically, at 2 mGy and 2 mm slice thickness, temperature tolerance spanned from 23 °C to 9.7 °C. With additional post-processing denoising, noise in physical density images decreased, and consequently, temperature tolerance at the same parameters decreased to 6.8 °C to 1.8 °C (figure 6(B)). As a result, the minimum radiation dose to meet the required temperature tolerance decreased to 2 mGy for each slice thickness compared to 57, 30, and 20 mGy without denoising at slice thicknesses of 2, 3, and 5 mm, respectively.

## Discussion

Spectral CT thermometry demonstrated reproducibility in liver-mimicking phantoms and achieved clinically relevant temperature tolerance by adjusting radiation dose and slice thickness. The incorporation of denoising further reduced the minimum radiation dose needed to meet clinical temperature accuracy requirements. These findings, including reproducibility, low temperature tolerance, and reduced radiation dose, support the clinical application of spectral CT for non-invasive temperature monitoring during liver tumor ablations.

Compared to conventional CT thermometry studies, this study not only utilized a direct model of thermal volumetric expansion but also implemented liver-mimicking phantoms, investigated the effect of the scan protocol on temperature tolerance, and applied post-processing to address the clinical requirements of spectral CT thermometry. Liver-mimicking phantoms were utilized for the assessment of reproducibility and temperature tolerance of spectral CT thermometry. While previous studies have used *ex vivo* and *in vivo* tissues of varying types (Paul *et al* 2015, Saccomandi *et al* 2016, Leng Lee *et al* 2019), these organ tissues are naturally heterogeneous and vary from sample to sample, both in terms of attenuation and tissue conductivity properties. Intra- and inter-sample variation of *ex vivo* tissue may thus hinder any evaluation of reproducibility as well as mask the effect of acquisition parameters on temperature tolerance by mixing heterogeneity with image noise (Liu *et al* 2023a). Liver-mimicking phantoms, on the other hand, are homogeneous and its synthesis repeatable to ensure it maintains the same properties from batch to batch. They also replicate the heating properties of liver but without the complex biology. Even so, there may be some variation that then translates to the CV of the slope, likely due to the use of three separate phantoms. Other potential sources of variation for CV include the lack of additional denoising, air bubbles at the tip of temperature probes, and the similar but inconsistent heating method between phantoms. Similarly, it is expected that the CV would also demonstrate variation in *ex vivo* and *in vivo* tissues as a result of inter-sample heterogeneity. Additionally, the experimental setup only included the phantom and did not represent other complex structures such as vasculature and vertebrae that would be present in clinical procedures. Future studies prior to clinical translation will require *in vivo* evaluation that accounts for tissue heterogeneity, the presence of other anatomical structures, and the heat-sink effect.

In addition to liver-mimicking phantoms, evaluation of spectral CT thermometry required a new methodology for calculating temperature accuracy specific to the model. This study utilized temperature tolerance, which is derived from error propagation of thermal volumetric expansion, as a surrogate of temperature accuracy. This methodology accounts for our recently introduced model as well as factors in the effect of noise present in CT images that may affect the accuracy of generated temperature maps. As a result, temperature tolerance in spectral CT thermometry is acquisition and reconstruction parameter dependent. To match the accuracy of previous thermometry studies, radiation doses between 10 and 30 mGy were required for slice thicknesses ranging from 2 to 5 mm. For even lower temperature tolerance, higher radiation doses were needed but are not optimal for clinical translation.

In order to reduce the radiation dose, additional denoising was implemented to ensure the clinical requirement for temperature accuracy was met. While the denoising here was performed on a piecewise constant phantom (Negussie *et al* 2016) and thus may not be practical for clinical translation, it provides an example of post processing that may significantly benefit the clinical translation of spectral CT thermometry. Another example is metal artifact reduction for ablation applicators. In this study, a water bath was utilized rather than an ablation applicator, which allowed for heating of the entire phantom slowly that ensures small temperature changes during the duration of a CT scan for the entire phantom. However, it did not mimic the clinical scenario where metal artifacts from the ablation applicator reduce image quality and quantitative integrity (Huang *et al* 2015) that would propagate to temperature maps, particularly in the vicinity of the applicator itself. As a result, quantitatively accurate metal artifact reduction is required for accurate and clinically useful non-invasive temperature monitoring. Such development and evaluation of metal artifact reduction algorithms for spectral CT thermometry are currently in progress and will ensure artifact-free and reliable temperature maps. The addition of both post-processing algorithms, denoising and metal artifact reduction, will likely have an impact on spatial resolution and will be evaluated in a future study utilizing phantoms with complex structures as well as *in vivo* pig models.

Reproducibility and clinically relevant temperature tolerance of spectral CT thermometry bolster its utility for non-invasive temperature monitoring to provide real-time feedback to interventional radiologists during thermal ablation. Unlike spectral CT thermometry, current methods require interventional radiologists to extrapolate between pre-ablation and post-ablation contrast-enhanced CT scans (visual/cognitive registration) to establish a complete ablation and sufficient minimal ablative margin (Wood *et al* 2007, Park *et al* 2008). Even with the addition of ablation confirmation software, follow-up magnetic resonance imaging still indicated that only 77% of ablations had a sufficient minimal ablative margin (Joo *et al* 2022), suggesting the need for other techniques. Specifically, spectral CT thermometry’s intraprocedural, volumetric temperature maps cannot only discern the tumor volume that has already reached the lethal threshold but also track temperatures of surrounding critical structures to minimize thermal damage. With such a tool and its information, interventional radiologists can adjust ablation duration in real-time to guarantee a complete ablation and a sufficient minimal ablative margin but requires confidence in spectral CT thermometry. Reproducibility of spectral CT thermometry builds this confidence by demonstrating thermal volumetric expansion and the same model parameters even with different phantoms. Additionally, both temperature accuracy and radiation dose requirement are met by adjusting acquisition parameters and applying additional post-processing. Each of these three aspects enhance spectral CT thermometry’s utility for non-invasive temperature monitoring in clinical thermal ablation procedures.

In conclusion, physical density maps demonstrated a strong, reproducible relationship with temperature in liver-mimicking phantoms with temperature sensitivities that varied with acquisition and reconstruction parameters. Temperature tolerance was demonstrated at clinically relevant levels and the required radiation dose was lowered to 2 mGy with denoising. By meeting the requirements for temperature tolerance and radiation dose, spectral CT thermometry can increasingly facilitate the clinical adoption of non-invasive temperature monitoring during thermal ablation procedures, thereby reducing the risk of local tumor recurrence.

## Data Availability

The data cannot be made publicly available upon publication because no suitable repository exists for hosting data in this field of study. The data that support the findings of this study are available upon reasonable request from the authors.
